# FKB327, an adalimumab biosimilar, versus the reference product: results of a randomized, Phase III, double-blind study, and its open-label extension

**DOI:** 10.1186/s13075-019-2046-0

**Published:** 2019-12-12

**Authors:** Mark C. Genovese, Josephine Glover, Maria Greenwald, Wieslawa Porawska, Elias Chalouhi El Khouri, Eva Dokoupilova, Juan Ignacio Vargas, Mykola Stanislavchuk, Herbert Kellner, Elena Baranova, Nobuhito Matsunaga, Rieke Alten

**Affiliations:** 1 Division of Immunology and Rheumatology, Stanford University, 1000 Welch Rd, #203, Palo Alto, CA USA; 2 Coephycient Pharmaceutical Consultancy, Guildford, UK; 3 Desert Medical Advances, Palm Desert, CA USA; 4 Centrum Badań Klinicznych S.C, Poznański Ośrodek Medyczny NOVAMED, Poznań, Poland; 5 Clínica Internacional, Lima, Peru; 6 MEDICAL PLUS s.r.o., Uherské Hradiště, Czech Republic; Faculty of Pharmacy, Department of Pharmaceutics, University of Veterinary and Pharmaceutical Sciences, Brno, Czech Republic; 7 Quantum Research, Puerto Varas, Chile; 8 National Pirogov Memorial Medical University, Vinnytsia, Ukraine; 9 Center for Rheumatology and Gastroenterology, Munich, Germany; 10 First Saint-Petersburg State Medical University, St. Petersburg, Russia; 11 Fujifilm Kyowa Kirin Biologics, Tokyo, Japan; 12 Schlosspark-Klinik, University Medicine Berlin, Berlin, Germany

**Keywords:** Rheumatoid arthritis, Adalimumab, Biosimilar, FKB327, Comparative clinical trials, Efficacy, Safety, Immunogenicity, Pharmacokinetics

## Abstract

**Objective:**

To compare the efficacy, serum drug concentrations, immunogenicity, and safety of FKB327 with the adalimumab reference product (RP) in combination with methotrexate in patients with moderate-to-severe, active rheumatoid arthritis (RA).

**Methods:**

Patients were randomized 1:1 in a double-blind study (NCT02260791), received 40 mg of FKB327 or RP by subcutaneous injection every other week for 24 weeks (Period I), then re-randomized 2:1, remaining on the same study drug or switching to the other up to week 54 in an open-label extension (Period II, NCT02405780). Efficacy was evaluated using American College of Rheumatology (ACR20) response rate difference at week 24 with equivalence margins of ± 13% and − 12% to + 15% using 95% and 90% confidence intervals (CIs), respectively. Efficacy, serum drug concentrations, immunogenicity, and safety were compared at week 54.

**Results:**

A total of 730 patients were randomized in Period I (*n* = 367 FKB327, *n* = 363 RP), and 645 transitioned to Period II (*n* = 216 FKB327–FKB327, *n* = 108 FKB327–RP, *n* = 108 RP–FKB327, *n* = 213 RP–RP). At week 24, ACR20 response rates were 74.1% with FKB327 versus 75.7% with RP. 95% and 90% CI of the response rate difference were − 7.9 to 4.7% and − 7.3 to 3.6%, respectively, meeting predefined equivalence margins. The ACR20 response rate remained over 70% of patients to week 54 with all treatment sequences. In Period I, mean trough serum drug concentrations were slightly higher for patients receiving FKB327 than those receiving RP. Mean concentrations were stable over time and reflected steady state in Period II. The proportions of patients with samples positive for neutralizing antidrug antibodies (ADAs) were comparable (57.7% with FKB327 vs. 55.5% with RP) at week 24, and no consistent difference in ADA were seen between continuous and switched treatments in Period II. Efficacy was slightly reduced in the small proportion of patients with high ADA titers in all treatment groups. No clinically significant differences were observed in the incidence of commonly reported treatment-emergent adverse events between the treatments across Periods I and II.

**Conclusion:**

FKB327 was equivalent to RP in clinical efficacy and demonstrated comparable safety and immunogenicity in patients with moderate-to-severe RA. No effect of switching between FKB327 and RP was observed.

**Trial registration:**

ClinicalTrials.gov, NCT02260791, Registered 29 July 2014.

ClinicalTrials.gov, NCT02405780, Registered 17 July 2015.

## Introduction

Rheumatoid arthritis (RA) is a chronic autoimmune disease characterized by joint pain, stiffness, swelling, and loss of function. The pro-inflammatory cytokine tumor necrosis factor (TNF)-α plays a key role in RA pathogenesis, and TNF-α inhibition has demonstrated positive clinical outcomes [1, 2]. The TNF-α inhibitor adalimumab (Humira®; AbbVie), hereafter referred to as the reference product (RP), is a biological disease-modifying anti-rheumatic drug (bDMARD) that has been approved for the treatment of patients with moderate-to-severe, active RA [3–5]. Currently, RP is licensed for use in combination with the conventional synthetic disease-modifying anti-rheumatic drug (DMARD) methotrexate (MTX) or as monotherapy in many other indications [5].

As existing licensed therapies are approaching or have passed their patent expiration, increasing numbers of TNF-α inhibitor biosimilars for RA therapy are currently in clinical development [6–11]. FKB327 is being developed as a proposed biosimilar product that contains adalimumab but has different excipients to those in RP, all of which are commonly used in biologic treatments. Preclinical studies have shown that FKB327 and RP are comparable with respect to binding to the target antigens (human recombinant TNF-α and transmembrane TNF-α), as well as inducing apoptosis and cellular cytotoxicity [12]. A subsequent Phase I clinical study of FKB327 and RP has demonstrated comparable pharmacokinetic, safety, and immunogenicity profiles [13].

The double-blind, Phase III study (Period I) and corresponding open-label extension study (Period II) were conducted to further compare the efficacy, safety, pharmacokinetics, and immunogenicity of FKB327 and RP. Antidrug antibodies (ADAs) can develop against biologic agents such as TNF-α inhibitors. Previous studies have demonstrated that ADAs can be associated with reduced response to treatment, the potential for increased risk of injection site reactions (ISR) and infections, and may lead to a reduction in serum drug concentrations [14]. Consequently, the effect of immunogenicity on the efficacy and safety of FKB327 was also evaluated.

## Patients and methods

### Patients

The double-blind study (Period I; NCT02260791) included patients aged ≥ 18 years with active RA diagnosed according to the revised American College of Rheumatology (ACR) criteria (2010 version) ≥ 3 months prior to screening. Active RA was confirmed by at least six out of 68 tender joints and at least six out of 66 swollen joints at screening and baseline, and a C-reactive protein (CRP) level ≥ 10 mg/L at screening. Inclusion criteria included taking MTX (oral or parenteral) for ≥ 3 months prior to screening at a stable dose of 10–25 mg/week for ≥ 8 weeks, with concomitant folic acid dose of ≥ 5 mg/week. Stable doses for ≥ 4 weeks were required if concomitant oral steroids (≤ 10 mg/day prednisone or equivalent) or non-steroidal anti-inflammatory drugs (NSAIDs) were taken.

The main exclusion criteria included prior treatment with RP or any adalimumab-containing biosimilar and/or more than one bDMARD or targeted synthetic DMARD (tsDMARD) for RA; prior treatment with a TNF inhibitor for RA with primary lack of efficacy as the reason for discontinuation; use of intra-articular or parenteral steroids or DMARDs other than MTX within 28 days or more of screening, depending on half-life; ACR functional Class IV; presence of chronic or acute infection at screening, including human immunodeficiency virus (HIV), hepatitis B, hepatitis C, and active or untreated latent tuberculosis (TB); or any active autoimmune disease or joint disease other than RA or malignancy in the 5 years prior to screening that had not been curatively treated (except for fully excised carcinoma in situ of the cervix or basal cell carcinoma).

For inclusion in the extension study (Period II; NCT02405780), patients were required to have completed all 24 weeks of the double-blind study (Period I), with a minimum of nine doses of study drug received, and continued stable, concomitant MTX and folate treatment. Patients should have shown an adequate clinical response according to the investigator.

### Study design

Period I was a multicenter, randomized, double-blind, Phase III, parallel-arm, active-comparator trial to demonstrate comparable efficacy and safety of FKB327 to RP. Sample size was calculated to show equivalence of the ACR20 response rate at week 24 on FKB327 and Humira with 80% power. Eligible patients were randomized 1:1, using an interactive web response system, to receive either FKB327 or RP each at a dose of 40 mg by subcutaneous injection every other week from week 0 (baseline) through week 22. FKB327 was drawn from a vial into a syringe. Each sterile vial was filled with 0.8 mL deliverable volume of 50 mg/mL FKB327, delivering a dose of 40 mg. It was formulated in monosodium glutamate, sorbitol, methionine, polysorbate 80, hydrochloric acid, and water for injection at pH 5.2. RP was provided in prefilled syringes. It was delivered as a 40 mg dose in 0.8 mL, formulated in citric acid monohydrate, dibasic sodium phosphate dihydrate, mannitol, monobasic sodium phosphate dihydrate, polysorbate 80, sodium chloride, sodium citrate, and water for injection. For Period I, the single doses of the study drugs were provided in identical boxes and prepared by an unblinded individual. FKB327 was drawn from a vial into the syringe provided and placed into a masking unit by an unblinded individual at the study site, while RP syringes were provided already inserted into an identical masking unit. The injection was performed by an unblinded nurse not otherwise involved in the study assessments. The masking units were opaque plastic boxes with a sliding portion, which allowed the plunger to be depressed and the dose to be administered without revealing the appearance of the syringe to the patients, in order to prevent bias. No accidental unblinding to physician or patient was reported.

Dynamic randomization was applied using prior biologic treatment for RA (yes/no) and screening disease activity (Disease Activity Score 28 based on CRP [DAS28-CRP] ≤ 5.1/> 5.1) as stratification factors in order to balance treatment allocation within strata and by investigational site. At week 24, eligible patients who completed Period I could transition without interruption into Period II, such that the last visit (at week 24) of Period I became the first visit of Period II. Patients who did not enter Period II had a follow-up visit at week 26.

The open-label extension study (Period II) was of novel design because of re-randomization between the investigational and reference treatments. Before starting treatment in Period II, patients were re-randomized so that two thirds remained on the same treatment that they had received in Period I, providing approximately 1 year (54 weeks) of continuous treatment and observations on both FKB327 and RP. The remaining patients were re-randomized to switch to the opposite treatment at the beginning of Period II. As during Period I, doses of 40 mg FKB327 or RP were administered subcutaneously every other week during Period II. In Period II, FKB327 and RP were self-administered using identical-appearing prefilled syringes.

### Endpoints and assessments

The primary endpoint of Period I (double-blind study) was the American College of Rheumatology (ACR20) response rate at week 24. Two sets of equivalence margins and confidence intervals (CIs) for the difference in the ACR20 response rate were applied in order to meet different requirements from regulatory authorities. Criterion 1 was an equivalence margin of ± 13% using 95% CI, and Criterion 2 was an equivalence margin of − 12 to + 15% using 90% CI based on advice received from EU Committee for Medicinal Products for Human Use and the US Food and Drug Administration, respectively.

Secondary endpoints of Period I included DAS28-CRP at week 24 (key secondary endpoint) with an equivalence margin of ± 0.6, and ACR20, ACR50, and ACR70 response rates over time. Trough serum drug concentrations were collected at week 0 and prior to dosing at weeks 2, 4, 12, 20, and 24, then summarized by treatment group. Serum concentrations of adalimumab were determined using a validated immunoassay on an electrochemiluminescence (ECL) platform with a lower limit of detection of 100 ng mL^− 1^. ADA activity blood samples were collected at week 0 and prior to dosing at weeks 2, 4, 12, and 24. A highly sensitive ECL bridging format (Meso Scale Discovery) with acid dissociation to increase drug tolerance was used for immunogenicity assessments, which included the proportion of ADA-positive patients in each group, and ADA titer. ADA titer was defined as low (less than or equal to the lower quartile), moderate (between the lower and upper quartile, both not included), or high (greater than or equal to the upper quartile). Subgroup analyses by ADA titer were conducted of ACR20 response rate, DAS28-CRP at weeks 24 and 54, and changes in DAS28-CRP from baseline. The effects of maximum ADA titer on serum drug concentrations in each treatment arm were analyzed over 54 weeks. The percentage of patients with neutralizing ADAs was assessed by sensitive competitive ligand binding. Since these were competitive ligand binding assays, they were able to detect new and additional ADAs throughout both study periods, even in patients with high titers.

Safety was assessed through the reporting of adverse events, physical examinations, vital sign measurements, electrocardiograms, chest X-rays, and clinical laboratory tests. Treatment-emergent adverse events (TEAEs) were summarized by system organ class and preferred term using the Medical Dictionary for Regulatory Activities. TEAEs of special interest were identified using the prescribing information for the RP [5]. The QuantiFERON®-TB Gold In-Tube test (Quest Diagnostics, Madison, NJ, USA) was used to detect TB infection. TB screening was performed at baseline and at weeks 22 and 48. Patients with a positive or indeterminate QuantiFERON®-TB Gold result for latent TB at baseline were permitted to enter the study provided they had a clear chest X-ray and had completed at least 3 weeks of prophylactic anti-TB treatment prior to study drug administration. Patients with a newly positive or indeterminate QuantiFERON®-TB Gold test result and negative chest X-ray were not permitted to continue on study treatment until at least 3 weeks of anti-TB prophylaxis were given. In some cases, the time taken for TB investigations and prophylactic treatment exceeded the maximum 4-week interruption of treatment permitted by the protocol and the patient had to be discontinued from the study.

The primary objective of Period II was to compare the long-term safety of FKB327 and RP, which was assessed as for Period I. An important secondary objective was to compare the effect of switching between treatments on efficacy, safety, and immunogenicity assessments.

### Statistical analysis

The full analysis set (FAS), used for efficacy analyses, was defined as patients who received at least one dose of treatment and who had at least one evaluable primary efficacy measurement. For the primary analysis, the 90% and 95% CIs for treatment difference in the ACR20 response rate at week 24 in Period I were calculated using a normal approximation method and missing values were imputed using root cause imputation and non-responder imputation. ACR20, ACR50, and ACR70 response rates were summarized using percentages and 95% CIs via the Clopper–Pearson method. DAS28-CRP was summarized using descriptive statistics. The 95% CI for treatment difference of DAS28-CRP at week 24 was calculated based on a mixed model for repeated measures with terms for stratification variables, site, visit, treatment, and treatment by visit interaction to demonstrate equivalence. For long-term efficacy evaluation in the extension study, descriptive statistics were calculated.

Patients were included in the serum drug concentration analysis set if they had received at least one dose of study treatment and had at least one serum drug concentration result after treatment. Trough serum drug concentrations were summarized using descriptive statistics. The safety analysis set (SAS), defined as patients who received at least one dose of treatment, was used for safety and immunogenicity analyses. Datasets and outputs were produced using SAS® version 9.1 or higher.

## Results

### Patients

**Fig. 1:**
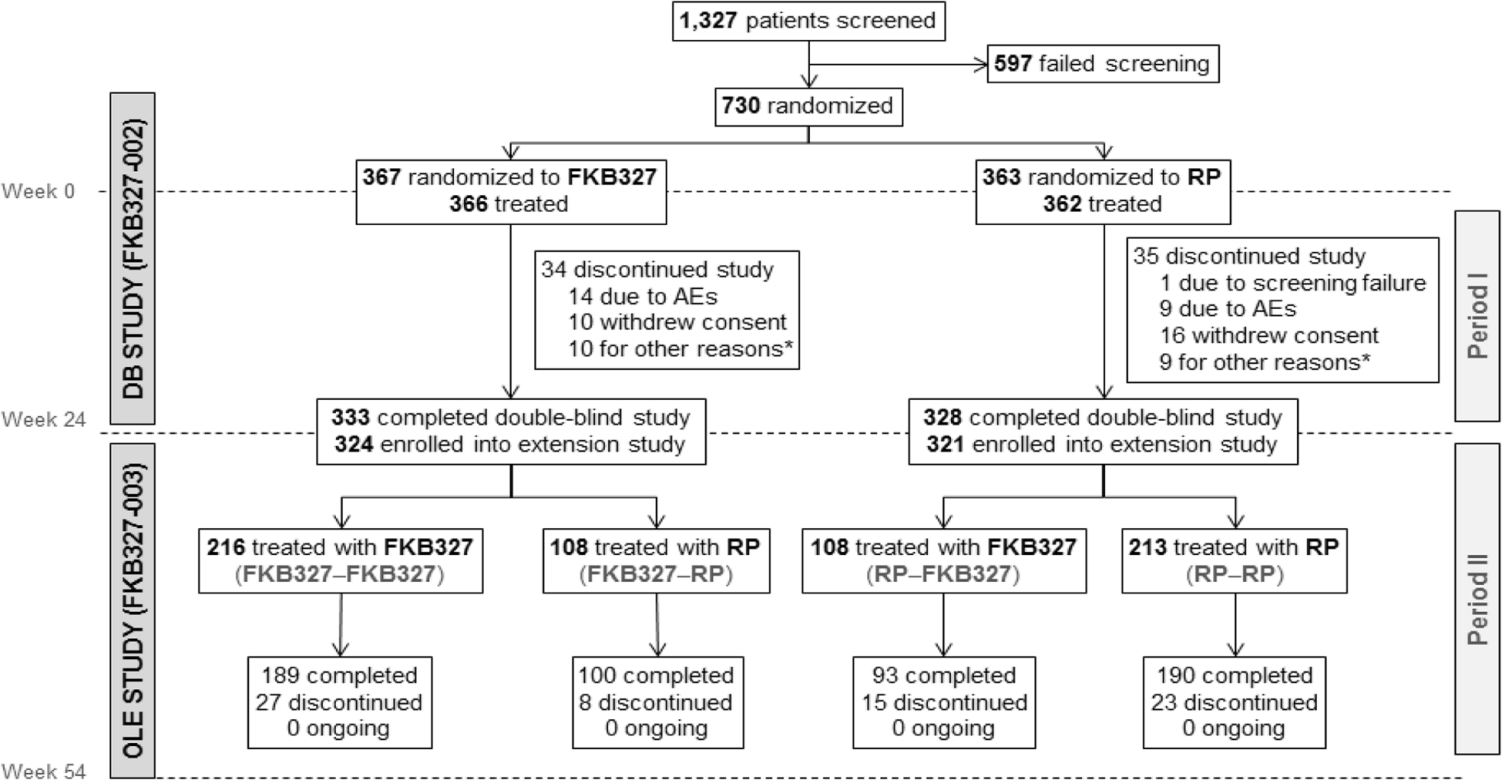
Patient disposition in Periods I and II (encompassing the double-blind and extension study). *AE* adverse event, *DB* double-blind, *OLE* open-label extension, *RP* reference product. *Including two patients treated with FKB327 and one patient treated with RP who discontinued study treatment due to lack of efficacy

Patients were recruited from 109 sites located in 12 countries (Bulgaria, Canada, Chile, Czech Republic, Germany, Peru, Poland, Romania, Russia, Spain, Ukraine, and the USA). In total, 1327 patients were screened for Period I: 730 patients were randomized, 728 patients received study treatment, and 597 patients failed screening (Fig. 1); of those 65.7% (167/597), patients were excluded due to a positive result for HIV, hepatitis B, hepatitis C, or TB.

A total of 645 patients (88.4% of the Period I study randomized population) entered Period II (the extension study): 324 patients (88.3%) in the FKB327 group and 321 patients (88.4%) in the RP group (Fig. 1).

Five-hundred and seventy-two patients (88.7%) completed Period II and 73 patients (11.3%) discontinued during that Period. In the SAS, 216 patients had received FKB327 in Period I and Period II, 108 patients received FKB327 followed by RP, 108 patients received RP followed by FKB327, and 213 patients received RP for both periods.

Baseline patient demographics and disease characteristics for Period I (Table 1) were generally well balanced between the treatment groups. Concomitant medication was kept stable throughout the study periods.

**Table 1:** Baseline patient demographics and disease characteristics for Period I

	FKB327 *n* = 366*	RP *n* = 362	Total *N* = 728^†^
Mean age (SD), years	53.0 (12.0)	53.6 (12.3)	53.3 (12.2)
Gender, *n* (%)			
Male	85 (23.2)	78 (21.5)	163 (22.4)
Female	281 (76.8)	284 (78.5)	565 (77.6)
Race, *n* (%)			
White	311 (85.0)	308 (85.1)	619 (85.0)
Black or African-American	2 (0.5)	4 (1.1)	6 (0.8)
Other^‡^	53 (14.5)	50 (13.8)	103 (14.1)
Mean disease duration (SD), years	8.6 (8.3)	8.3 (7.6)	8.5 (8.0)
Rheumatoid factor status, *n* (%)			
Positive	277 (75.7)	277 (76.5)	554 (76.1)
Negative	88 (24.0)	83 (22.9)	171 (23.5)
Missing	1 (0.3)	2 (0.6)	3 (0.4)
Mean DAS28-CRP (SD)	6.1 (0.9)	6.1 (0.9)	6.1 (0.9)
Mean CRP level (SD), mg/L	25.0 (26.7)	26.6 (28.4)	25.8 (27.6)
Mean tender joint count (68-joint count; SD)	26.2 (14.5)	25.9 (14.5)	26.1 (14.5)
Mean swollen joint count (66-joint count; SD)	16.2 (9.1)	16.0 (9.0)	16.1 (9.0)
Mean patient assessment of disease activity (SD)	68.0 (17.9)	68.2 (18.2)	68.1 (18.0)
Mean physician assessment of disease activity (SD)	68.4 (14.6)	66.4 (15.0)	67.4 (14.8)
Mean patient assessment of pain (SD)	66.7 (18.7)	67.9 (18.6)	67.3 (18.6)
Mean Health Assessment Questionnaire score (SD)	1.8 (0.5)	1.8 (0.5)	1.8 (0.5)
Prior medication for RA			
At least one biologic, *n* (%)	65 (17.8)	67 (18.5)	132 (18.1)
At least one DMARD,^§^ *n* (%)	236 (64.5)	229 (63.3)	465 (63.9)
At least one TNF inhibitor, *n* (%)	22 (6.0)	27 (7.5)	49 (6.7)
Concomitant medication for RA			
Mean MTX dose (SD), mg/week	15.8 (5.0)	15.8 (4.6)	15.8 (4.8)
At least one oral steroid and at least one NSAID, *n* (%)	137 (37.4)	149 (41.2)	286 (39.3)

*n = 365 for CRP level, tender joint count, swollen joint count, patient assessment of disease activity, patient assessment of pain, and Health Assessment Questionnaire; *n* = 364 for DAS28-CRP and physician assessment of disease activity

^†^*n* = 727 for CRP level, tender joint count, swollen joint count, patient assessment of disease activity, patient assessment of pain and Health Assessment Questionnaire; *n* = 726 for DAS28-CRP and physician assessment of disease activity

^‡^Includes the categories American Indian or Alaska Native (*n* = 2), Asian (*n* = 2), and other (*n* = 99)

^§^Both biologic and non-biologic DMARDs were included

*CRP* C-reactive protein, *DAS28-CRP* disease activity score 28 based on C-reactive protein, *DMARD* disease-modifying anti-rheumatic drug, *MTX* methotrexate, *NSAID* non-steroidal anti-inflammatory drug, *RA* rheumatoid arthritis, *RP* reference product, *SD* standard deviation, *TNF* tumor necrosis factor

Patient demographics for Period II (Additional file 3: Table S1) were generally balanced, although a lower proportion of patients aged ≥ 65 years received the RP–FKB327 treatment sequence (11.1%) compared with RP–RP (20.7%). There were minor imbalances in baseline disease characteristics across the four treatment sequences, which may have been due to the smaller patient numbers per group compared with Period I.

### Efficacy

**Fig. 2:**
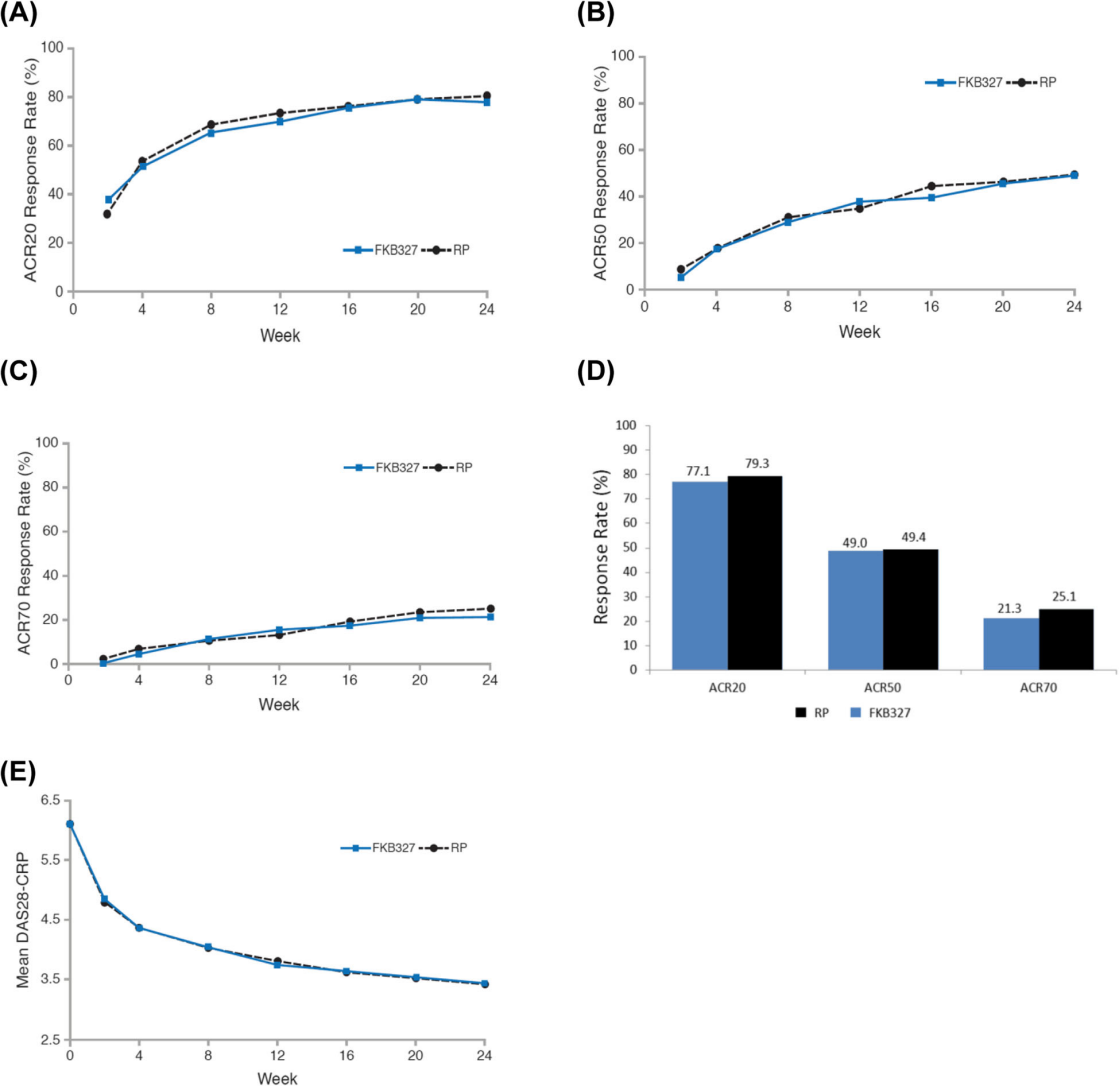
Efficacy results from Period I (double-blind study) from weeks 0 to 24 for **a** ACR20 response rate, **b** ACR50 response rate, **c** ACR70 response rate, **d** ACR20, ACR50, and ACR70 response rates at week 24, **e** mean DAS28-CRP (95% CI) from weeks 0 to 24. *ACR* American College of Rheumatology, *CI* confidence interval, *DAS28-CRP* disease activity score 28 based on C-reactive protein, *RP* reference product

During Period I, nine patients (1.2%) were excluded from the FAS because they either did not receive a study drug or did not have a primary efficacy measurement after the first dose. Efficacy analyses, therefore, included 721 patients (363 in the FKB327 group and 358 in the RP group). At week 24, 74.1% (*n* = 269/363) of the FKB327 group achieved an ACR20 response compared with 75.7% (*n* = 271/358) of the RP group. The 95% CI for treatment difference (FKB327–RP) was − 7.9 to 4.7. The 90% CI for treatment difference was − 7.3 to 3.6. The least squares mean (LSM) DAS28-CRP at week 24 was 3.43 on FKB327 and 3.42 on RP, with the 95% CI of − 0.16 to 0.18. All CI values fell within their predefined equivalence margins. Mean DAS28-CRP over time was comparable for both treatment groups (Fig. 2e).

The proportion of patients with an ACR20 response was comparable between the treatment groups from week 2 to week 24 (Fig. 2a). ACR50 and ACR70 response rates were also comparable throughout Period I (Fig. 2b, c). The proportions of patients achieving ACR20, ACR50, and ACR70 response at week 24 were comparable for the two treatments (Fig. 2d). Subgroup analyses of ACR20 response rate at week 24 by the stratification factors of prior biologic treatment for RA (yes/no), screening DAS28-CRP (≤ 5.1/> 5.1), and geographic region (North America/Europe/rest of world) showed no differences between the treatment groups (Additional file 1: Figure S1).

**Fig. 3:**
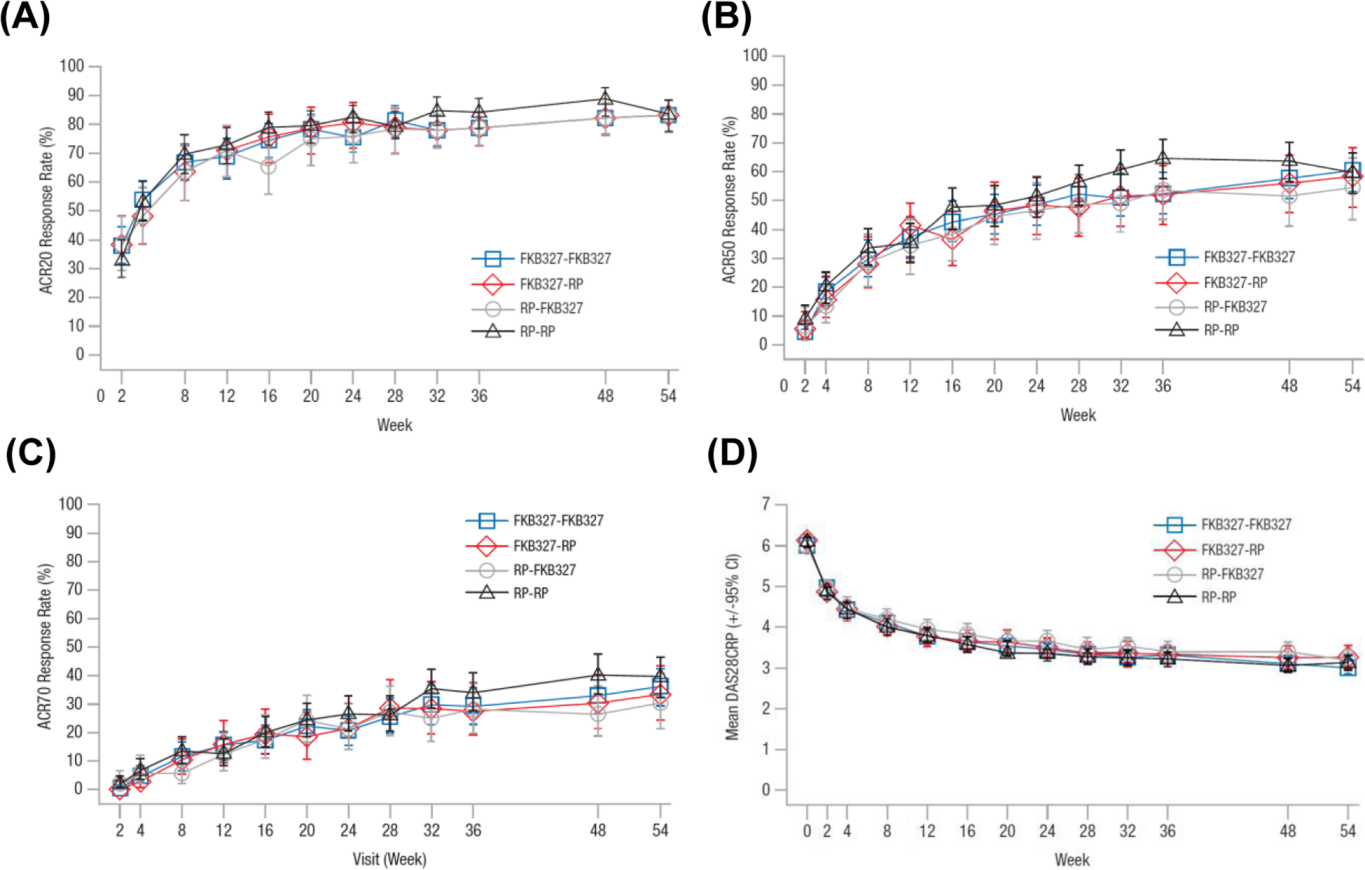
Long-term efficacy results from weeks 0 to 54 of treatment (to the end of Period II); **a** ACR20 response rate, **b** ACR50 response rate, **c** ACR70 response rate, **d** mean DAS28-CRP (95% CI). *ACR* American College of Rheumatology, *CI* confidence interval, *DAS28-CRP* disease activity score 28 based on C-reactive protein, *RP* reference product

During Period II, no patients were excluded from the FAS, and efficacy was analyzed for 645 patients (Fig. 1). There was a slight imbalance in efficacy measures between the four treatment sequences at the beginning of Period II due to a lack of stratification on re-randomization. However, efficacy was maintained throughout Period II, irrespective of treatment switching, with comparable ACR20, ACR50, and ACR70 response rates and mean DAS28-CRP over time on all four treatment sequences (Fig. 3).

### Trough serum drug concentrations

In Period I, analyses of trough serum drug concentrations were conducted for 722 patients: 364 in the FKB327 group and 358 in RP group. The mean serum trough drug concentration–time profiles were broadly comparable between the two treatment groups. Geometric LSM serum trough concentrations at week 24 were slightly higher on FKB327 than RP (4124.3 ng/mL and 3761.7 ng/mL, respectively). The ratio of the geometric LSM (90% CI) trough serum adalimumab concentrations at week 24 (FKB327:RP) was 1.10 (0.95, 1.27), and the 90% CIs for this ratio at all sampling points included unity.

At baseline in Period II (631 patients) mean trough serum drug concentrations were slightly higher in the treatment sequences that had received FKB327 in Period I. Interindividual variability in systemic exposure was high, with coefficients of variation of 60.7 to 78.2% across the four treatment sequences. Mean concentrations were stable over time and reflected steady state. Geometric LSM serum trough concentrations averaged over all time points in Period II (weeks 36, 48, and 54) for all patients receiving FKB327 and RP in this period were 4396.4 ng/mL and 4106.6 ng/mL and the ratio (FKB327:RP) of the geometric LSMs (90% CI) averaged over all time points was 1.07 (0.93, 1.23), which included unity.

### Immunogenicity

ADA analyses were conducted for the SAS (*n* = 728). The proportion of ADA-positive patients in each treatment group was comparable at each time point. At week 24, there were 212 (57.9%) and 201 (55.5%) ADA-positive patients in the FKB327 and RP groups, respectively. The distribution of ADA titer results at the last sampling time point was comparable in the two treatment groups; there were no differences in the proportion of patients at each positive ADA titer level. At week 24, the proportions of patients with samples that tested positive for neutralizing ADAs were comparable for both treatments (57.7% with FKB327 vs. 55.5% with RP) and indicated that almost all ADAs detected were neutralizing. At week 54, the proportions of ADA-positive patients were 52.2%, 61.0%, 45.2%, and 51.6%, respectively, for the FKB327-FKB327, FKB–RP, RP–FKB327, and RP–RP treatment sequences (Additional file 4: Table S2). The slight differences between treatment groups were not considered significant given the smaller numbers of patients per group after re-randomization. The mean ADA titer was higher at week 54 for all treatment sequences compared with week 0, influenced by a small number of individuals; however, median ADA titer did not increase over time for any of the groups. A negative impact of ADA formation on serum drug concentration and efficacy was shown to be related to ADA titer.

**Fig. 4:**
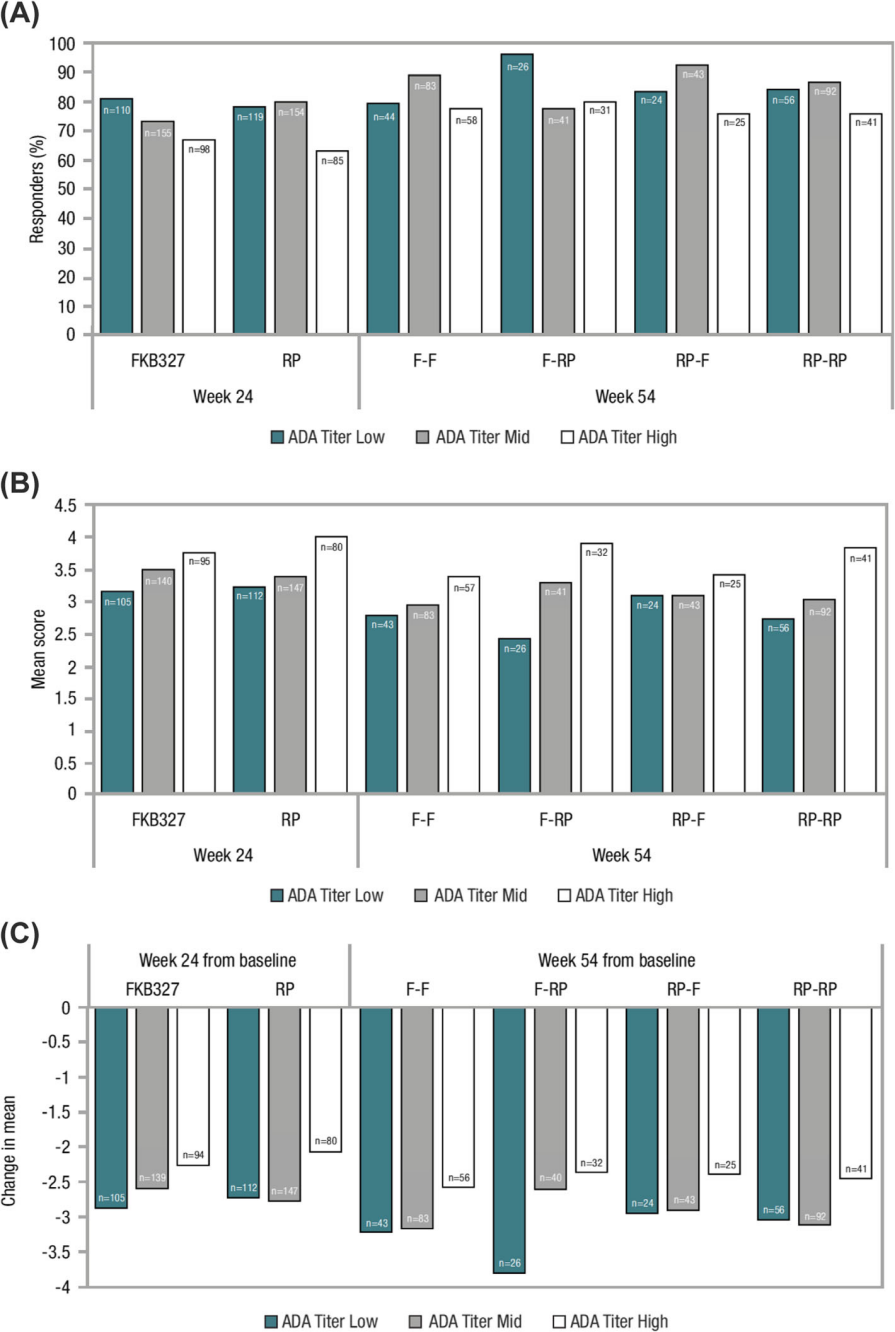
Effect of maximum ADA titer results on ACR20 response rate and mean DAS28-CRP at week 24 (end of Period I) and week 54 (Period II); **a** ACR20 response rate, **b** mean DAS28-CRP, **c** change in mean DAS28-CRP. *ACR* American College of Rheumatology, *ADA*, antidrug antibody, *DAS28-CRP* disease activity score 28 based on C-reactive protein, *F* FKB327, *RP* reference product. Low ADA titer was defined as ≤ the lower quartile, high ADA titer as ≥ the upper quartile, with mid titer between the lower and upper quartiles (both not included)

Low ADA titer was defined as ≤ the lower quartile, high ADA titer as ≥ the upper quartile, with mid titer between the lower and upper quartiles (both not included). Only a small proportion of patients fell into the high ADA titer category (low ADA titer *n* = 167, mid titer *n* = 291, high titer *n* = 172). Those with a high ADA titer experienced greater suppression of serum drug concentration than those with lower titer or negative status, but the same scale of effect was seen with FKB327 and RP (Additional file 2: Figure S2). High ADA titer and neutralizing ADA-positive status had a slight impact on ACR20 response rate and DAS28-CRP score throughout the study, with no additional loss of efficacy seen over time (Fig. 4). Since only a small proportion of patients developed high ADA titers, these findings were not reflected in the efficacy results for the overall study population described above. ADA titer was not shown to be related to incidence of potential hypersensitivity events, which occurred infrequently on both products.

### Safety

#### TEAEs

Table 2 shows an integrated summary of TEAEs reported in ≥ 3% of patients across Periods I and II. Percentages are based on the number of patients in Period I SAS who received treatment in either Period I or II. Hence, patients who switched at week 24 are included in the *N* count for both treatments. FKB327 was comparable to RP with respect to the overall safety profile. The most commonly reported TEAEs on both treatments were infections. In Period II, there were no clinically significant differences observed in the incidence of commonly reported TEAEs between the treatment sequences.

**Table 2:** Integrated summary of TEAEs and most common TEAEs across Periods I and II

	FKB327	RP
	*n* = 474	*n* = 470
	*n* (%)*	*n* (%)*
Summary of TEAEs		
Patients with at least one TEAE	295 (62.2)	311 (66.2)
Patients with at least one severe TEAE	17 (3.6)	12 (2.6)
Patients with at least one treatment-related TEAE	122 (25.7)	132 (28.1)
Patients with TEAE leading to treatment discontinuation	28 (5.9)	21 (4.5)
Patients with TEAE leading to treatment interruption	56 (11.8)	62 (13.2)
Patients with at least one TESAE	25 (5.3)	34 (7.2)
Deaths	2 (0.4)	1 (0.2)
Most common TEAEs (≥ 3% of patients in any group)^†^		
Blood and lymphatic system disorders	26 (5.5)	25 (5.3)
Anemia	14 (3.0)	13 (2.8)
Gastrointestinal disorders	48 (10.1)	55 (11.7)
Diarrhea	12 (2.5)	18 (3.8)
General disorders and administration site conditions	27 (5.7)	32 (6.8)
Infections and infestations	151 (31.9)	170 (36.2)
Nasopharyngitis	36 (7.6)	46 (9.8)
Upper respiratory tract infection	18 (3.8)	26 (5.5)
Bronchitis	16 (3.4)	27 (5.7)
Urinary tract infection	24 (5.1)	17 (3.6)
Pharyngitis	14 (3.0)	15 (3.2)
Latent tuberculosis	15 (3.2)	8 (1.7)
Injury, poisoning and procedural complications	27 (5.7)	31 (6.6)
Investigations	54 (11.4)	40 (8.5)
Metabolism and nutrition disorders	39 (8.2)	35 (7.4)
Hypercholesterolemia	20 (4.2)	16 (3.4)
Musculoskeletal and connective tissue disorders	67 (14.1)	65 (13.8)
RA	26 (5.5)	23 (4.9)
Nervous system disorders	29 (6.1)	34 (7.2)
Renal and urinary disorders	23 (4.9)	23 (4.9)
Respiratory, thoracic, and mediastinal disorders	21 (4.4)	20 (4.3)
Skin and subcutaneous tissue disorders	40 (8.4)	36 (7.7)
Vascular disorders	23 (4.9)	27 (5.7)
Hypertension	12 (2.5)	21 (4.5)

*Percentages are based on the number of patients in the double-blind study safety set who received the given treatment in either Period I or Period II. Hence, patients who switch at week 24 are included in the *N* count for both treatments

^†^Each patient was counted only once for each condition

*RA* rheumatoid arthritis, *RP* reference product, *TEAE* treatment-emergent adverse event, *TESAE* treatment-emergent serious adverse event

Treatment-emergent serious AEs (TESAEs) were experienced by 25 patients (5.3%) treated with FKB327 and 34 patients (7.2%) treated with RP up to the end of Period II. The most commonly reported TESAEs were infections (*n* = 13 [2.7%] on FKB327 vs. *n* = 10 [2.1%] on RP). Disseminated TB was reported for one patient in each treatment group and pulmonary TB in two patients on RP during Period I, but rigorous monitoring of regular TB testing results meant that no active TB was reported in Period II, despite the high endemic prevalence of TB in the recruiting countries. Three patients on FKB327 at the time of diagnosis developed malignancies (squamous cell carcinoma of the skin, myeloma, and cervical carcinoma), compared with two patients treated with RP (squamous cell carcinoma of the skin and lymphoma). A total of three deaths occurred during Periods I and II. In Period I, one patient on FKB327 died from disseminated TB. During Period II, one patient receiving RP–FKB327 (recently diagnosed with carcinoma of the cervix) died suddenly of unknown cause and one patient on RP–RP died from a severe cerebrovascular accident.

The incidence of TEAEs leading to permanent discontinuation of treatment, mostly infections or diagnosis of latent TB on protocol-required testing, was comparable between the FKB327 and RP groups across Periods I and II (*n* = 28 [5.9%] vs. 21 [4.5%], respectively) and was not affected by switching treatments.

#### TB

At screening, the majority of patients had a negative QuantiFERON®-TB Gold test result (89.0%). At week 22, in the FKB327 arm, 35 patients (9.6%) had a positive QuantiFERON®-TB Gold test result and four (1.1%) had an indeterminate result. In the RP arm, 30 patients (8.3%) had a positive result, while six (1.7%) had an indeterminate result. The proportion of patients with a negative result at screening who had developed a positive or indeterminate QuantiFERON®-TB Gold test result was similar in the FKB327 and RP treatment groups (*n* = 26 [8.0%] vs. *n* = 20 [6.2%] respectively). There were three discontinuations due to latent TB on FKB327 and four discontinuations due to latent TB on RP.

#### ISR

The ISR was low and comparable between FKB327 and RP. At week 0 of Period I, 95.9 and 95.8% of patients on FKB327 and RP, respectively, experienced no irritation; 2.8 and 3.6%, respectively, reported minimal erythema; 1.1 and 0.3% respectively reported definite erythema; and 0.3% reported erythema with papules in both arms. The injection site pain visual analogue scale (VAS) score was also low and comparable between FKB327 and RP (mean [standard deviation]; 9.3 [14.83] vs. 20.2 [23.16]). Similarly, at week 24, 94.4 and 95.6% of patients experienced no ISR irritation with FKB327 and RP, respectively; 5.3 and 4.1% reported minimal erythema, respectively; and 0.3% reported definite erythema in both arms. The injection site pain VAS score was low and comparable between FKB327 and RP (mean [standard deviation]; 6.2 [9.70] vs. 12.9 [18.80]).

## Discussion

The double-blind study (Period I) met its primary objective, demonstrating equivalence in efficacy of FKB327 and RP. Both the 95% and 90% CIs for ACR20 response rates at week 24 fell well within the prespecified equivalence margins and are comparable to those previously reported for RP when compared with placebo and with concomitant MTX [15].

In the extension study (Period II), there was no evidence that switching between treatments caused a significant change in efficacy, immunogenicity or safety compared with maintaining the same treatment received in Period I.

The safety profile for patients treated with FKB327 across both studies was comparable with that of those treated with RP, and consistent with the known risks of the latter. Of note, latent TB was one of the most reported AEs on both products; however, this was deemed to be due to the high incidence of TB in the general population of some of the countries included in the study (Peru, Poland, Romania, Russia, and Ukraine) and regular testing required by the study protocols.

The proportion of ADA-positive patients was much higher than seen in previous studies with RP where < 1% (2/207) and 1.5% (1/69) of patients dosed with 40 mg every other week and 1.4% (1/73) of patients dosed with 80 mg every other week were shown to be ADA positive [15, 16]. This is most likely due to the use of a considerably more sensitive assay technique than used in earlier RP studies, namely solid-phase extraction with acid dissociation. Our findings suggest that the majority of patients will develop ADAs to RP, even with immune suppression from concomitant MTX [17] and that most of these will be neutralizing. The presence of a high proportion of neutralizing ADA-positive patients would be expected to have negative implications for efficacy due to reduced serum drug concentrations [18, 19]. In this study, it has been shown that the impact of ADAs on serum drug concentration and efficacy is related to ADA titer for both drugs, and only a small proportion of patients developed a high ADA titer within the extended study period. No additional effect of high ADA titer on efficacy measures was seen between weeks 24 (the time point at which maximum ADA-positive rate was seen) and 54. Thus ACR20 response rate and DAS28-CRP scores in the study population as a whole remained stable in the long term on both treatments and despite switching treatments.

Furthermore, hypersensitivity events associated with immunogenicity did not occur at higher rates than in earlier studies despite the higher proportion of ADA-positive patients detected. This minimal impact on efficacy and safety suggests that the high rates of detection of ADA positivity for both FKB327 and RP are not clinically important [20].

## Conclusion

In conclusion, the Phase III double-blind study met its primary and overall objectives by demonstrating comparability of FKB327 and RP in terms of efficacy, safety, and immunogenicity results. No effect of switching between FKB327 and RP treatments was observed in the extension study, and overall, these results support the proposal of FKB327 as a candidate biosimilar to RP in patients with active RA.

## Supplementary information

**Additional file 1:** **Figure S1.** Subgroup analysis of ACR20 response rate at week 24 (end of Period I) by (A) prior biological treatment for RA and screening DAS28-CRP category and (B) geographical region.

**Additional file 2:** **Figure S2.** Effect of maximum ADA titer results on serum drug concentration from weeks 0 to 54 for (A) FKB327–FKB327, (B) FKB327–RP, (C) RP–FKB327 and (D) RP–RP.

**Additional file 3:** **Table S1**. Baseline patient demographics and disease characteristics by treatment sequence in Period II.

**Additional file 4:** **Table S2.** ADA status at scheduled sampling points during Period II (extension study).

## Data Availability

The datasets used and/or analyzed during the current study are available from the corresponding author on reasonable request.

## Abbreviations

ACR: American College of Rheumatology
ADA: Antidrug antibody
bDMARD: Biological disease-modifying anti-rheumatic drug
CI: Confidence interval
CRP: C-reactive protein
DAS28-CRP: Disease Activity Score 28 based on C-reactive protein
DMARD: Disease-modifying anti-rheumatic drug
ECL: Electrochemiluminescence
FAS: Full analysis set
MTX: Methotrexate
NSAID: Non-steroidal anti-inflammatory drug
RA: Rheumatoid arthritis
RP: Reference product
SAS: Safety analysis set
TB: Tuberculosis
TEAE: Treatment-emergent adverse event
TNF: Tumor necrosis factor

## References

[1] Choy E (2012). Understanding the dynamics: pathways involved in the pathogenesis of rheumatoid arthritis. Rheumatology (Oxford).

[2] Smolen JS, Landewé R, Bijlsma J, Burmester G, Chatzidionysiou K, Dougados M (2017). EULAR recommendations for the management of rheumatoid arthritis with synthetic and biological disease-modifying antirheumatic drugs: 2016 update. Ann Rheum Dis.

[3] US Food & Drug Administration. BLA application number 125057: approval letter. https://www.accessdata.fda.gov/drugsatfda_docs/nda/2002/BLA_125057_S000_HUMIRA_APPROV.PDF. Accessed 1 Aug 2019.

[4] AbbVie Inc. Humira® summary of product characteristics. http://www.ema.europa.eu/docs/en_GB/document_library/EPAR_-_Product_Information/human/000481/WC500050870.pdf. Accessed 1 Aug 2019.

[5] AbbVie Inc. Humira® prescribing information. http://www.rxabbvie.com/pdf/humira.pdf. Accessed 15 Aug 2019.

[6] Weinblatt ME, Baranauskaite A, Dokoupilova E, Zielinska A, Jaworski J, Racewicz A (2018). Switching from reference adalimumab to SB5 (adalimumab biosimilar) in patients with rheumatoid arthritis: fifty-two-week phase 3 randomized study results. Arthritis Rheumatol.

[7] Weinblatt ME, Baranauskaite A, Niebrzydowski J, Dokoupilova E, Zielinska A, Jaworski J (2018). Phase III randomized study of SB5, an adalimumab biosimilar, versus reference adalimumab in patients with moderate-to-severe rheumatoid arthritis. Arthritis Rheumatol.

[8] Jamshidi A, Gharibdoost F, Vojdanian M, Soroosh SG, Soroush M, Ahmadzadeh A (2017). A phase III, randomized, two-armed, double-blind, parallel, active controlled, and non-inferiority clinical trial to compare efficacy and safety of biosimilar adalimumab (CinnoRA®) to the reference product (Humira®) in patients with active rheumatoid arthritis. Arthritis Res Ther.

[9] Jani RH, Gupta R, Bhatia G, Rathi G, Ashok Kumar P, Sharma R (2016). A prospective, randomized, double-blind, multicentre, parallel-group, active controlled study to compare efficacy and safety of biosimilar adalimumab (Exemptia; ZRC-3197) and adalimumab (Humira) in patients with rheumatoid arthritis. Int J Rheum Dis.

[10] Cohen S, Genovese MC, Choy E, Perez-Ruiz F, Matsumoto A, Pavelka K (2017). Efficacy and safety of the biosimilar ABP 501 compared with adalimumab in patients with moderate to severe rheumatoid arthritis: a randomised, double-blind, phase III equivalence study. Ann Rheum Dis.

[11] Cohen SB, Alonso-Ruiz A, Klimiuk PA, Lee EC, Peter N, Sonderegger I (2018). Similar efficacy, safety and immunogenicity of adalimumab biosimilar BI 695501 and Humira reference product in patients with moderately to severely active rheumatoid arthritis: results from the phase III randomised VOLTAIRE-RA equivalence study. Ann Rheum Dis.

[12] European Medicines Agency. Human medicine European public assessment report: Hulio. https://www.ema.europa.eu/en/documents/assessment-report/hulio-epar-public-assessment-report_en.pdf. Accessed 25 Nov 2019.

[13] Puri A, Niewiarowski A, Arai Y, Nomura H, Baird M, Dalrymple I (2017). Pharmacokinetics, safety, tolerability and immunogenicity of FKB327, a new biosimilar medicine of adalimumab/Humira, in healthy subjects. Br J Clin Pharmacol.

[14] Pecoraro V, De Santis E, Melegari A, Trenti T (2017). The impact of immunogenicity of TNFalpha inhibitors in autoimmune inflammatory disease. A systematic review and meta-analysis. Autoimmun Rev.

[15] Weinblatt ME, Keystone EC, Furst DE, Moreland LW, Weisman MH, Birbara CA (2003). Adalimumab, a fully human anti-tumor necrosis factor alpha monoclonal antibody, for the treatment of rheumatoid arthritis in patients taking concomitant methotrexate: the ARMADA trial. Arthritis Rheumatol.

[16] Keystone EC, Kavanaugh AF, Sharp JT, Tannenbaum H, Hua Y, Teoh LS (2004). Radiographic, clinical, and functional outcomes of treatment with adalimumab (a human anti-tumor necrosis factor monoclonal antibody) in patients with active rheumatoid arthritis receiving concomitant methotrexate therapy: a randomized, placebo-controlled, 52-week trial. Arthritis Rheumatol.

[17] Krieckaert CL, Nurmohamed MT, Wolbink GJ (2012). Methotrexate reduces immunogenicity in adalimumab treated rheumatoid arthritis patients in a dose dependent manner. Ann Rheum Dis.

[18] Mok CC, Tsai WC, Chen DY, Wei JCC (2016). Immunogenicity of anti-TNF biologic agents in the treatment of rheumatoid arthritis. Expert Opin Biol Ther.

[19] Schaeverbeke T, Truchetet M-E, Kostine M, Barnetche T, Bannwarth B, Richez C (2016). Immunogenicity of biologic agents in rheumatoid arthritis patients: lessons for clinical practice. Rheumatology (Oxford).

[20] Song S, Yang L, Trepicchio WL, Wyant T (2016). Understanding the supersensitive anti-drug antibody assay: unexpected high anti-drug antibody incidence and its clinical relevance. J Immunol Res.

