# Supplementary material for: FKB327, an adalimumab biosimilar, versus the reference product: results of a randomized, Phase III, double-blind study, and its open-label extension
Source: Arthritis Res Ther. 2019 Dec 12;21:281. doi: 10.1186/s13075-019-2046-0 (PMC6909638; doi:10.1186/s13075-019-2046-0)
Supplement: Supplementary file 1 — Additional file 1: Figure S1. Subgroup analysis of ACR20 response rate at week 24 (end of Period I) by (A) prior biological treatment for RA and screening DAS28-CRP category and (B) geographical region. [file 13075_2019_2046_MOESM1_ESM.docx]

**Fig. S1** Subgroup analysis of ACR20 response rate at Week 24 (end of Period I) by (A) prior biological treatment for RA and screening DAS28-CRP category and (B) geographical region


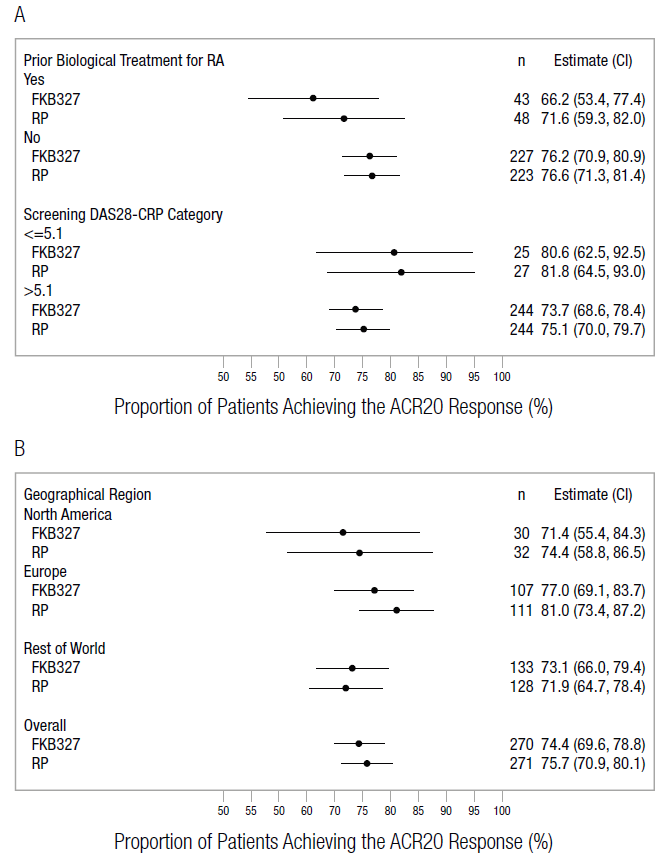


*ACR* American College of Rheumatology, *CI* confidence interval, *DAS28-CRP* disease activity score 28 based on C-reactive protein, *RA* rheumatoid arthritis, *RP* reference product.
