# Supplementary material for: FKB327, an adalimumab biosimilar, versus the reference product: results of a randomized, Phase III, double-blind study, and its open-label extension
Source: Arthritis Res Ther. 2019 Dec 12;21:281. doi: 10.1186/s13075-019-2046-0 (PMC6909638; doi:10.1186/s13075-019-2046-0)
Supplement: Supplementary file 2 — Additional file 2: Figure S2. Effect of maximum ADA titer results on serum drug concentration from weeks 0 to 54 for (A) FKB327–FKB327, (B) FKB327–RP, (C) RP–FKB327 and (D) RP–RP. [file 13075_2019_2046_MOESM2_ESM.docx]

**Fig. S2** Effect of maximum ADA titer results on serum drug concentration from Weeks 0 to 54 for (**A**) FKB327–FKB327, (**B**) FKB327–RP, (**C**) RP–FKB327 and (**D**) RP–RP

**(A)**


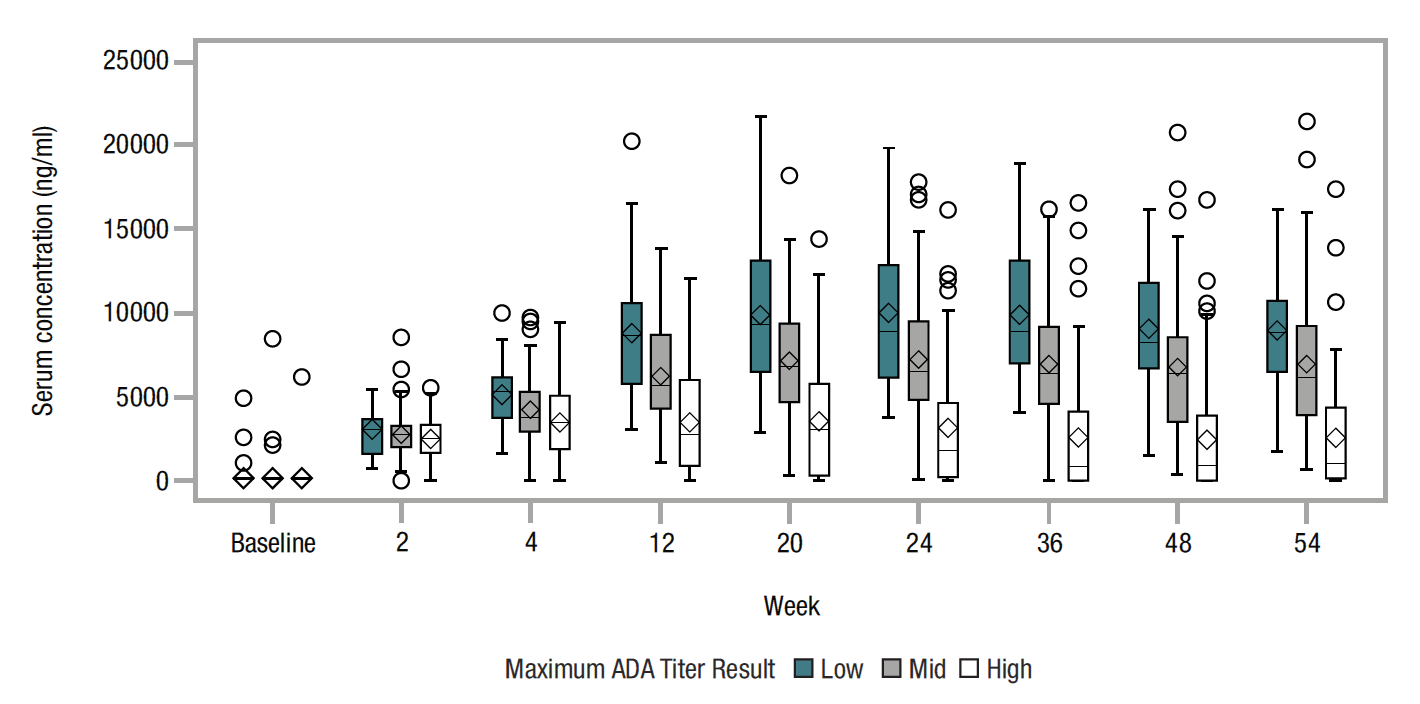


**(B)**


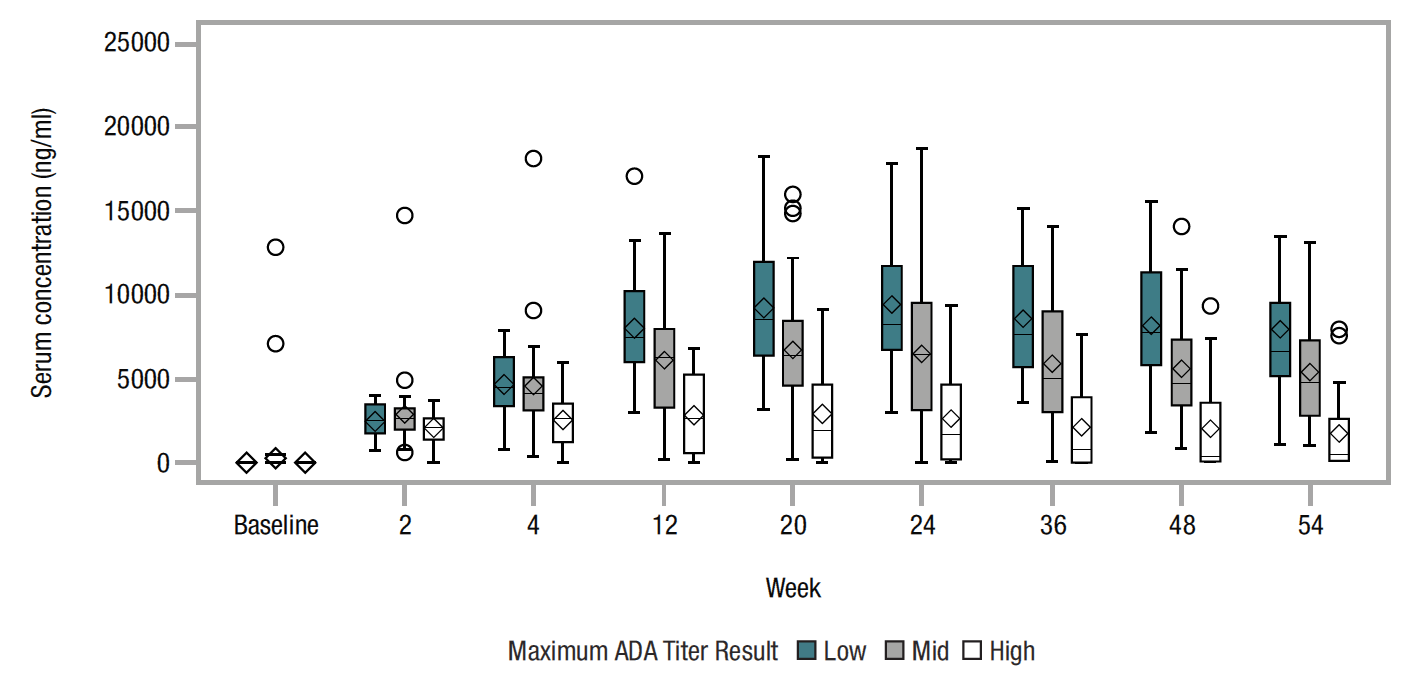


**(C)**
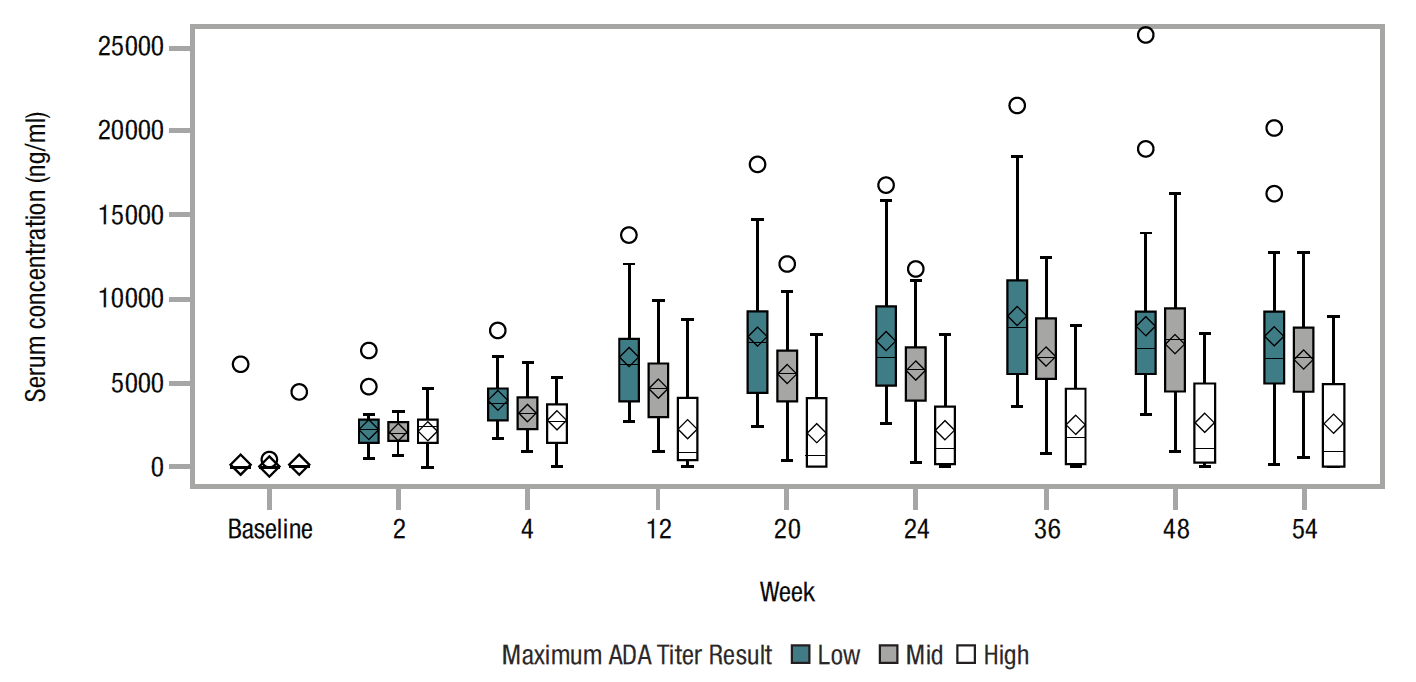


**(D)**


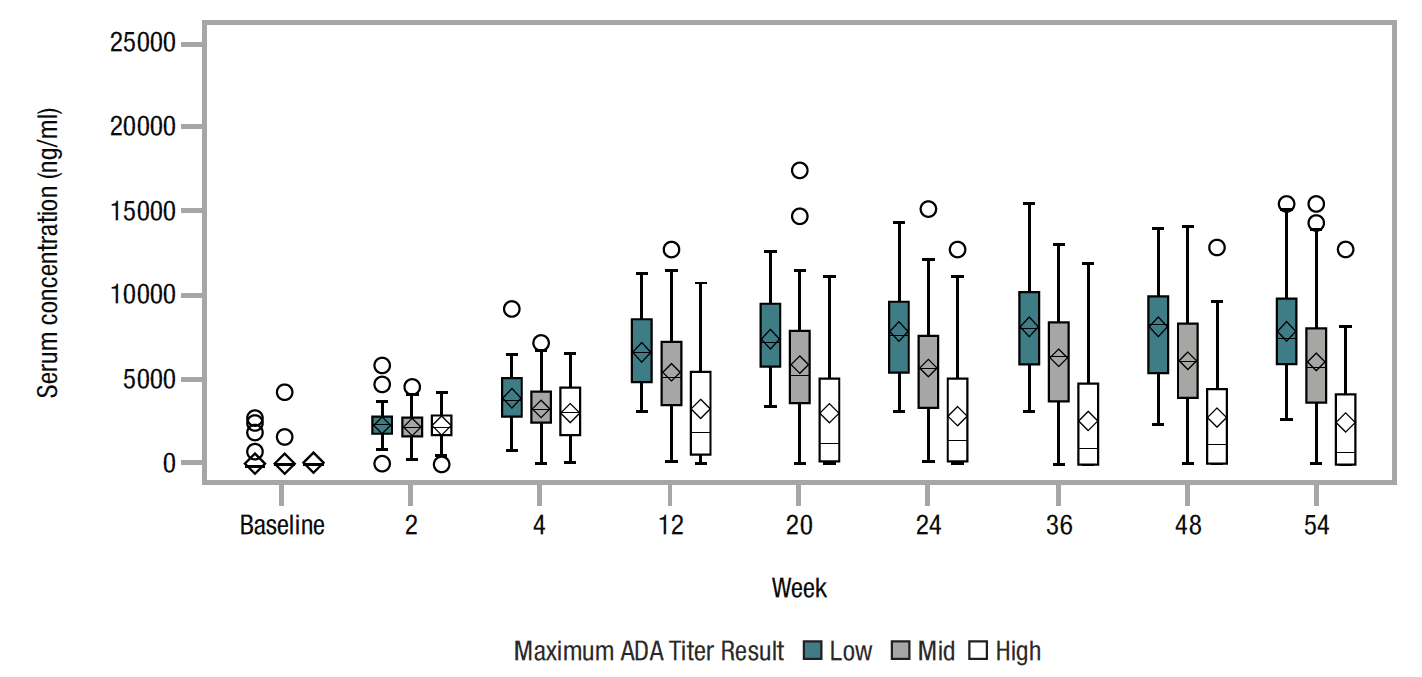


*ADA* antidrug antibody, *RP*, reference product. Low ADA titer was defined as ≤ the lower quartile, high ADA titer as ≥ the upper quartile, with mid titer between the lower and upper quartiles (both not included)
