# Supplementary material for: FKB327, an adalimumab biosimilar, versus the reference product: results of a randomized, Phase III, double-blind study, and its open-label extension
Source: Arthritis Res Ther. 2019 Dec 12;21:281. doi: 10.1186/s13075-019-2046-0 (PMC6909638; doi:10.1186/s13075-019-2046-0)
Supplement: Supplementary file 3 — Additional file 3: Table S1. Baseline patient demographics and disease characteristics by treatment sequence in Period II. [file 13075_2019_2046_MOESM3_ESM.docx]

**Table S1** Baseline patient demographics and disease characteristics by treatment sequence in Period II

|  | **FKB327–FKB327**  ***n* = 216*** | **FKB327–RP**  ***n* = 108** | **RP–FKB327**  ***n* = 108** | **RP–RP**  ***n* = 213^†^** | **Total**  ***N* = 645** |
| --- | --- | --- | --- | --- | --- |
| Mean age (SD), years | 52.7 (12.35) | 52.1 (11.35) | 52.3 (11.93) | 54.0 (12.60) | 52.9 (12.20) |
| Gender, n (%)  Male  Female | 54 (25.0)  162 (75.0) | 23 (21.3)  85 (78.7) | 25 (23.1)  83 (76.9) | 42 (19.7)  171 (80.3) | 144 (22.3)  501 (77.7) |
| Race, n (%)  White  Black or African American  American Indian or Alaska Native  Asian  Other | 187 (86.6)  1 (0.5)  1 (0.5)  1 (0.5)  26 (12.0) | 90 (83.3)  1 (0.9)  0  0  17 (15.7) | 90 (83.3)  2 (1.9)  0  1 (0.9)  15 (13.9) | 185 (86.9)  2 (0.9)  1 (0.5)  0  25 (11.7) | 552 (85.6)  6 (0.9)  2 (0.3)  2 (0.3)  83 (12.9) |
| Rheumatoid factor status, n (%)  Positive  Negative  Missing | 164 (75.9)  52 (24.1)  0 | 82 (75.9)  25 (23.1)  1 (0.9) | 85 (78.7)  23 (21.3)  0 | 159 (74.6)  52 (24.4)  2 (0.9) | 490 (76.0)  152 (23.6)  3 (0.5) |
| Mean DAS28-CRP (SD) | 3.5 (1.29) | 3.5 (1.31) | 3.7 (1.35) | 3.4 (1.32) | 3.5 (1.31) |
| Mean CRP level (SD), mg/L | 9.7 (13.81) | 13.7 (21.40) | 12.0 (19.20) | 11.5 (18.75) | 11.4 (17.85) |
| Mean tender joint count (68 joint count; SD) | 8.5 (10.61) | 8.6 (10.50) | 8.7 (9.11) | 7.8 (9.57) | 8.3 (10.0) |
| Mean swollen joint count (66 joint count; SD) | 3.5 (5.15) | 4.3 (7.47) | 4.2 (6.13) | 3.0 (4.64) | 3.6 (5.63) |
| Mean patient assessment of disease activity (SD) | 36.5 (24.27) | 33.8 (23.40) | 36.1 (22.60) | 31.4 (23.24) | 34.3 (23.57) |
| Mean physician assessment of disease activity (SD) | 21.8 (17.82) | 20.3 (16.31) | 22.1 (16.49) | 20.2 (15.99) | 21.0 (16.74) |
| Mean patient assessment of pain (SD) | 36.1 (24.35) | 32.6 (22.51) | 35.2 (23.53) | 32.3 (23.46) | 34.1 (23.63) |
| Mean Health Assessment Questionnaire score (SD) | 1.19 (0.676) | 1.27 (0.731) | 1.32 (0.678) | 1.23 (0.732) | 1.24 (0.705) |
| *Prior medication for RA* |  |  |  |  |  |
| At least one biologic, n (%) | 34 (15.7) | 21 (19.4) | 22 (20.4) | 39 (18.3) | 116 (18.0) |
| At least one DMARD,^§^ n (%) | 141 (65.3) | 70 (64.8) | 74 (68.5) | 133 (62.4) | 418 (64.8) |
| At least one TNF inhibitor, n (%) | 14 (6.5) | 4 (3.7) | 7 (6.5) | 16 (7.5) | 41 (6.4) |
| *Concomitant medication for RA* |  |  |  |  |  |
| Mean MTX dose (SD), mg/week | 16.2 (5.16) | 15.5 (4.90) | 16.2 (4.62) | 15.7 (4.61) | 15.9 (4.85) |
| At least one oral steroid and at least one NSAID, n (%) | 77 (35.6) | 51 (47.2) | 49 (45.4) | 91 (42.7) | 268 (41.6) |

**n* = 215 for CRP level and DAS28-CRP.

^†^ *n* = 212 for physician assessment of disease activity, patient assessment of pain, and Health Assessment Questionnaire.

^§^Both biologic and non-biologic DMARDs were included.

*CRP* C-reactive protein; *DAS28-CRP* Disease Activity Score 28 based on C-reactive protein; *DMARD* disease modifying anti-rheumatic drug; *NSAID* non-steroidal anti-inflammatory drug; *RA* rheumatoid arthritis; *RP* reference product; *SD* standard deviation; *TNF* tumor necrosis factor.
