# Supplementary material for: FKB327, an adalimumab biosimilar, versus the reference product: results of a randomized, Phase III, double-blind study, and its open-label extension
Source: Arthritis Res Ther. 2019 Dec 12;21:281. doi: 10.1186/s13075-019-2046-0 (PMC6909638; doi:10.1186/s13075-019-2046-0)
Supplement: Supplementary file 4 — Additional file 4: Table S2. ADA status at scheduled sampling points during Period II (extension study). [file 13075_2019_2046_MOESM4_ESM.docx]

**Table S2** ADA status at scheduled sampling points during Period II (extension study)

|  | **FKB327–FKB327**  ***n* = 216** | **FKB327–RP**  ***n* = 108** | **RP–FKB327**  ***n* = 108** | **RP–RP**  ***n* = 213** |
| --- | --- | --- | --- | --- |
| Period II, Week 24  Total  Positive | 216  133 (61.6) | 108  69 (63.9) | 108  67 (62.0) | 212  123 (58.0) |
| Week 36  Total  Positive | 202  109 (54.0) | 103  60 (58.3) | 103  54 (52.4) | 202  102 (50.5) |
| Week 48  Total  Positive | 197  100 (50.8) | 100  58 (58.0) | 96  47 (49.0) | 199  101 (50.8) |
| Week 54  Total  Positive | 186  97 (52.2) | 100  61 (61.0) | 93  42 (45.2) | 190  98 (51.6) |

*ADA* antidrug antibody; *RP* reference product.
